# Supplementary material for: Fluorometric Detection of SARS-CoV-2 Single-Nucleotide Variant L452R Using Ligation-Based Isothermal Gene Amplification
Source: Bioengineering (Basel). 2023 Sep 23;10(10):1116. doi: 10.3390/bioengineering10101116 (PMC10603929; doi:10.3390/bioengineering10101116)
Supplement: Supplementary file 1 [file bioengineering-10-01116-s001.zip › bioengineering-2583706-supplementary.pdf]

Table S1. Oligonucleotides sequences (5′ to 3′) used in the assay.

	Name	Sequence (5′ → 3′)	Size (nt)
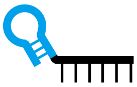	Hairpin probe (L452R)*	p-**GGT AAT TAT AAT TAC CAC CAA CCT TAG**<br>**AT**G ACC CTC CGT TTT TTT TTT TTT TTC<br>GGA GGG TC	62
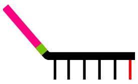	Linear probe (L452R)**	GAC CCT CCC CCC CAC TCC CTC AGC **AGA**<br>**CTT CCT AAA CAA TCT ATA CC**	47
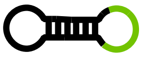	Dumbbell padlock***	p-GCA CGC CTA CTA **CCC TAA CCC TAA CCC**<br>**TAA CCC** TGC GTG CCG TAA CCC TAC TAC<br>ACC ACC ACC AC **G ACC CTC CCC CCC ACT**<br>**CCC TCA** GGT TAC G	94
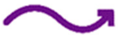	model SDAP	**TGA GG G AGT GGG GGG GAG GGT C**	22
	Wild-type /L452R RNA****	UCU AAG GUU GGU GGU AAU UAU AAU UAC<br>C**U/G**G UAU AGA UUG UUU AGG AAG UCU AA	53

*Hairpin probe: annealing site to both wild type and mutant RNA (black-colored and bold-faced sequences); hairpin probe loop (blue-colored sequences); hairpin probe stem (blue-colored underlined sequences): p, phosphorylation at the 5′-end.

** Linear probe: annealing sites to both wild type and mutant RNA (black-colored and bold-faced sequences), mutant RNA (pink-colored sequences) and single nucleotide variants of L452R (red-colored and yellow-highlighted sequence)

*** Dumbbell padlock: annealing site to the RCA primers generated by SDA (purple-colored and bold-faced sequences); C-rich sequences to generate the G-quadruplex (light green-colored and bold-faced sequences); p, phosphorylation at the 5′-end.

**** Wild type/L452R model RNA: wild type and mutant type sequence, respectively (Underlined and bold-faced).
